# Genotype-guided conservative management of mesenteric desmoid tumors: A case report of intermediate-region APC mutations

**DOI:** 10.1097/MD.0000000000047577

**Published:** 2026-02-20

**Authors:** Niu Huang, Guang Shi, Xue-lai Luo

**Affiliations:** a Department of Gastrointestinal Surgery, The People’s Hospital of Tongcheng, Tongcheng, Hubei Province, China; b Department of Gastrointestinal Surgery, Tongji Hospital, Tongji Medical College, Huazhong University of Science and Technology, Wuhan, Hubei Province, China.

**Keywords:** active surveillance, APC mutation, case report, desmoid tumor, genotype–phenotype correlation

## Abstract

**Rationale:**

Desmoid tumors (DTs) exhibit highly variable behavior, making management challenging. Specific adenomatous polyposis coli (APC) gene mutation sites are recognized as key prognostic markers, potentially enabling genotype-guided strategies to avoid overtreatment.

**Patient concerns:**

We present 2 symptomatic patients with familial adenomatous polyposis-associated mesenteric DTs. Patient 1 was a 46-year-old female with a large, symptomatic pelvic mass. Patient 2 was a 26-year-old male with multifocal recurrent disease, including a symptomatic abdominal wall lesion.

**Diagnoses:**

Diagnosis was confirmed by imaging and histopathology. Genetic sequencing identified intermediate-region APC mutations: a somatic c.1821T > A (p.Cys607Ter) mutation in patient 1 and a c.3183_3187delACAAA (p.Gln1062Ter) mutation in patient 2.

**Interventions:**

Management was stratified by genotype. Given the indolent-predicting mutations, patient 1 was managed with active surveillance alone. For patient 2, the symptomatic abdominal wall lesion was resected, and low-intensity systemic therapy (tamoxifen and celecoxib) was initiated for residual mesenteric disease.

**Outcomes:**

At 5-year follow-up, patient 1’s tumor showed >50% volume reduction with symptom alleviation. Patient 2 achieved sustained disease stability in all lesions at 3-year follow-up, with partial symptom remission. No significant treatment-related adverse events occurred.

**Lessons:**

Intermediate-region APC mutations (e.g., codons 607 and 1062) predict an indolent course in mesenteric DTs. Comprehensive APC genotyping at diagnosis enables risk-adapted management, permitting safe use of conservative strategies (active surveillance/low-intensity therapy) and helps avoid unnecessary aggressive interventions. This underscores the critical role of molecular profiling in personalizing DT care.

## 1. Introduction

Desmoid tumor (DT), or desmoid-type fibromatosis, is a rare, locally invasive fibroblastic proliferative lesion. It features recurrent potential without metastasis, but may recur locally.^[1,2]^ The disease was first described by MacFarlane in 1832.^[3]^ Global incidence is 5 to 6 per million,^[4]^ predominantly in women aged 30 to 40 years (male-to-female ratio: 1:2–3; median onset: 37–39 years).^[5,6]^ Mesenteric DTs are frequently associated with familial adenomatous polyposis (FAP).^[7]^ In FAP patients, DTs occur in 5% to 10% of cases, mostly intra-abdominal or abdominal wall, with mesenteric DTs being common.^[8]^ Classified anatomically as abdominal wall, intra-abdominal, or extra-abdominal.^[9]^ We report 2 mesenteric DT cases to investigate clinical features, diagnostic/therapeutic strategies, and mutation-based molecular mechanisms.

## 2. Patients and methods

**Figure 1.**
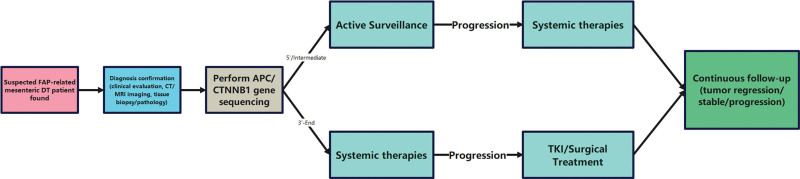
Patient flow diagram. CT = computed tomography, DT = desmoid tumor, FAP = familial adenomatous polyposis, MRI = magnetic resonance imaging, TKI = tyrosine kinase inhibitor.

We conducted a retrospective review of patients with mesenteric DTs associated with FAP who were managed at our institution between January 2020 and 2025. The 2 cases presented in this report were specifically selected because they harbored intermediate-region adenomatous polyposis coli (APC) gene mutations (codons 607 and 1062, respectively), which is the focus of this study, and they had complete clinical, radiological, and genetic data available for analysis. The diagnosis of DT was confirmed in both cases through a combination of contrast-enhanced computed tomography (CT) imaging, histopathological examination of biopsy or surgical specimens, and immunohistochemical staining. CT was the primary imaging modality for diagnosis and follow-up in both cases. Although magnetic resonance imaging provides superior soft tissue contrast, CT was utilized due to its lower cost, greater accessibility, and proven adequacy for evaluating tumor size, vascular relationships, and detecting complications such as obstruction or ischemia – key parameters for monitoring in a conservative management protocol. APC genetic sequencing was performed on tumor tissue as part of the standard diagnostic workup. Data regarding patient demographics, clinical presentation, management strategies, and outcomes were collected from electronic medical records. This study was approved by the Institutional Review Board of Tongcheng County People’s Hospital, and written informed consent was obtained from both patients. The overall diagnostic and genotype-guided management pathway is summarized in Figure 1.

## 3. Case reports

### 3.1. Case 1

A 46-year-old woman with a FAP history presented with an abdominal mass lasting >3 months. Past history included rectal cancer resection 12 years prior (with adjuvant chemotherapy) and regular intestinal polypectomy via high-frequency electrocautery.

**Figure 2.**
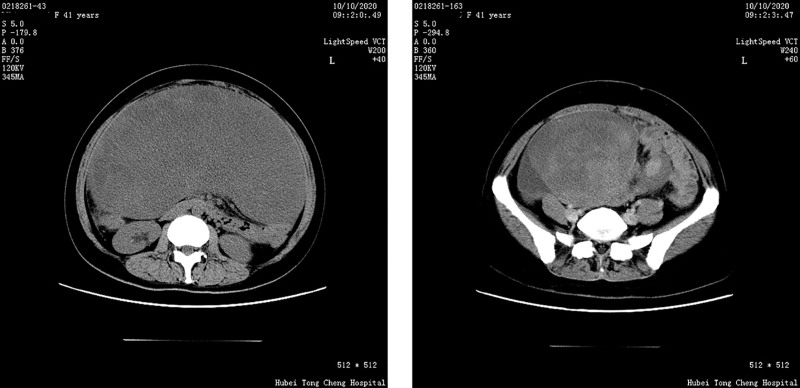
Preoperative CT image of patient in Case 1. CT = computed tomography.

**Figure 3.**
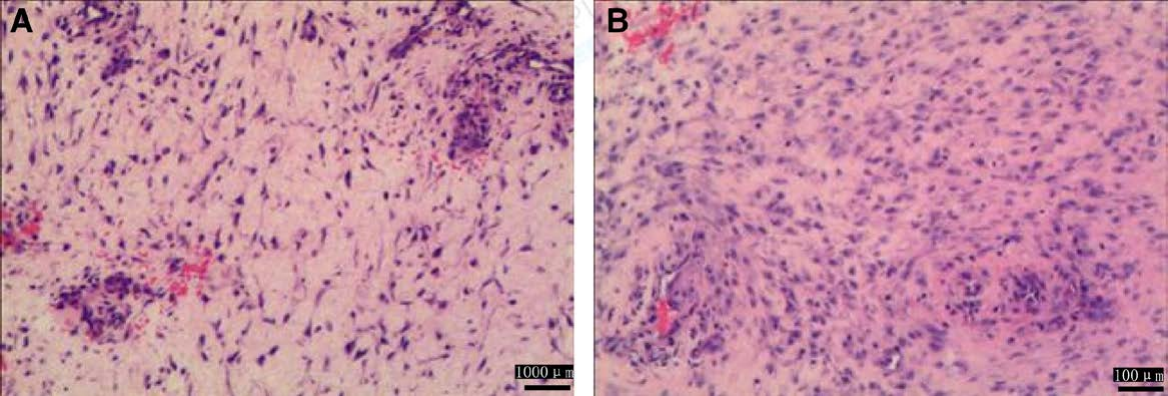
Histopathological analysis of the mesenteric mass in Case 1 (hematoxylin and eosin stain). (A) Low-power view (scale bar = 1000 μm) showing the characteristic bland, sweeping bundles of spindle-shaped fibroblasts within a collagenous stroma, consistent with desmoid-type fibromatosis. (B) High-power view (scale bar = 100 μm) highlighting the uniform, cytologically benign nuclei and infiltrative growth pattern at the tumor border (arrows). Note: These images were retrieved from the hospital’s electronic medical record system, and their resolution reflects the standard archival quality at the time of diagnosis.

**Figure 4.**
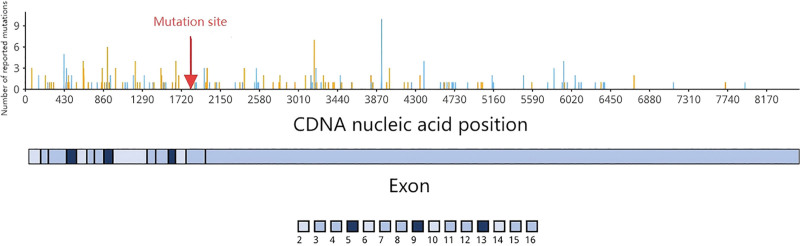
Sanger sequencing chromatogram of the APC gene in Case 1. The arrow indicates the heterozygous nonsense mutation c.1821T > A (p.Cys607Ter) in the tumor DNA. APC = adenomatous polyposis coli, DNA = deoxyribonucleic acid.

Abdominal CT revealed a large pelvic mass (126.5 × 252.5 mm) displacing adjacent organs. The mass demonstrated heterogeneous attenuation on precontrast images (25–37 Hounsfield Units) and marked heterogeneous enhancement post-contrast (58–75 Hounsfield Units), a degree of enhancement comparable to that of major pelvic vessels (Fig. 2). Critical to management decisions, the tumor was noted to encase >180° of the superior mesenteric artery and vein. Notably, there were CT features suggestive of non-occlusive mesenteric ischemia (NOMI), including bowel wall thickening and edema, likely resulting from direct vascular compression or a “steal phenomenon” by the hypervascular tumor. Core biopsy suggested DT. Given the large size and complex relationship with major mesenteric vessels, a core needle biopsy was deemed potentially risky. Therefore, a diagnostic laparotomy was performed, which confirmed a hypervascular mesenteric mass encircling the superior mesenteric vessels. Given the high morbidity of radical resection, only a representative biopsy was taken to establish a definitive histopathological diagnosis (Fig. 3). The decision to avoid aggressive surgery at this stage was strategically made to prevent surgical trauma that could potentially stimulate tumor growth. Immunochemistry: β-catenin (+), SMA (focal +), DES (focal +), CD117/CD34/DOG1/S-100/SOX10/STAT6/PCK (−), SDHB (+), MDM2 (±), P16 (+), CDK4 (−), and Ki-67 ~ 2%. Genetic sequencing revealed a heterozygous APC nonsense mutation c.1821T > A (p.Cys607Ter) (Fig. 4), causing premature termination at codon 607 and truncation via nonsense-mediated mRNA decay) . Findings confirmed invasive fibromatosis.

#### 3.1.1. Follow-up

**Figure 5.**
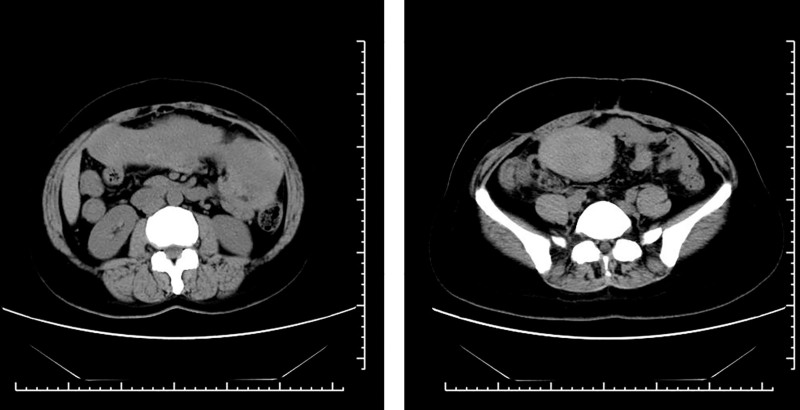
Case 1 patient’s follow-up CT image 5 years post-diagnosis (managed with active surveillance). cDNA = complementary deoxyribonucleic acid, CT = computed tomography.

**Figure 6.**
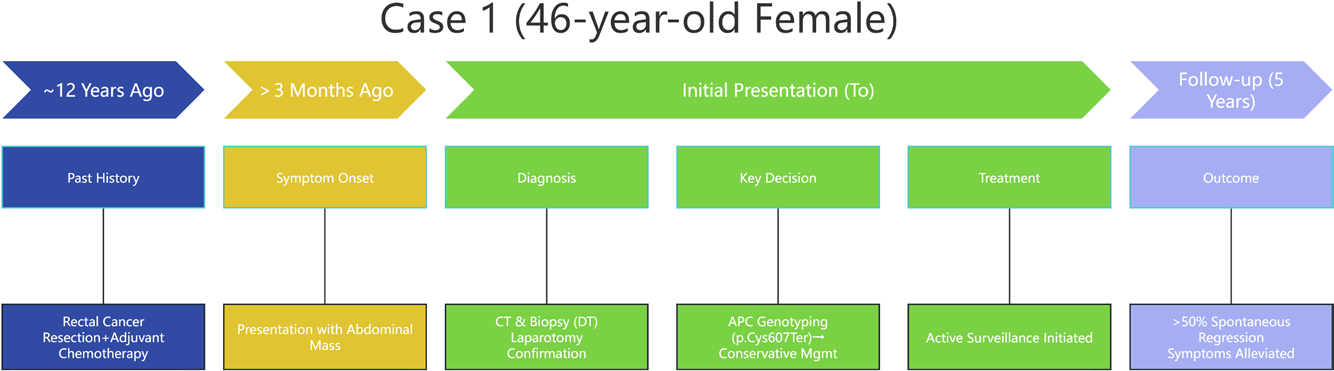
Timeline of the disease course in Case 1. CT = computed tomography, DT = desmoid tumor.

Given the identification of an APC mutation at codon 607 (intermediate region), which is associated with an indolent clinical course, the patient was managed with active surveillance alone. This management plan was discussed and confirmed by our institutional multidisciplinary tumor board. She was followed with clinical assessment and contrast-enhanced CT scans at 3-month intervals for the first year, then every 6 months thereafter. Tumor response was assessed using RECIST criteria (version 1.1; Elsevier Ltd., Amsterdam, The Netherlands), with regression defined as a ≥30% decrease in the sum of diameters and stability defined as a <20% increase or <30% decrease. At a 5-year follow-up, CT showed >50% tumor shrinkage and reduced organ compression (Fig. 5). Concomitantly, the previously noted signs of mesenteric ischemia had resolved completely. This clinical course was consistent with an indolent APC codon 607 mutation. Symptoms partially alleviated. Colonoscopies revealed no malignant polyps. The timeline of the disease course has been listed (Fig. 6).

### 3.2. Case 2

A 26-year-old man with a FAP history presented with a left lower abdominal wall mass lasting 1 year. History included total colectomy (6 years prior) and abdominal wall DT resection with adjuvant radiotherapy (5 years prior).

**Figure 7.**
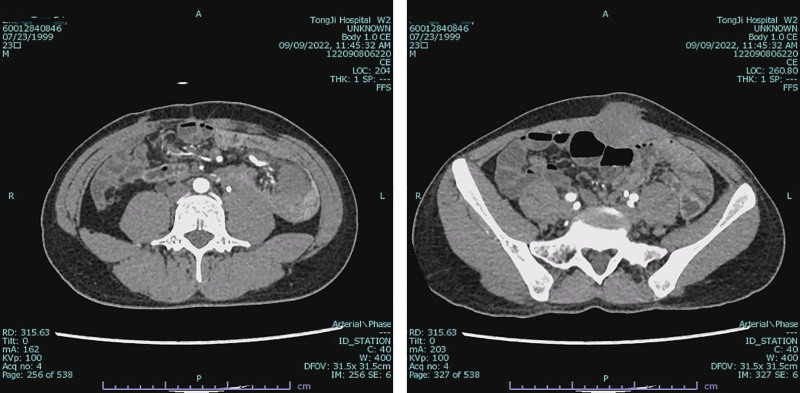
Preoperative CT image of patient in Case 2. CT = computed tomography.

**Figure 8.**
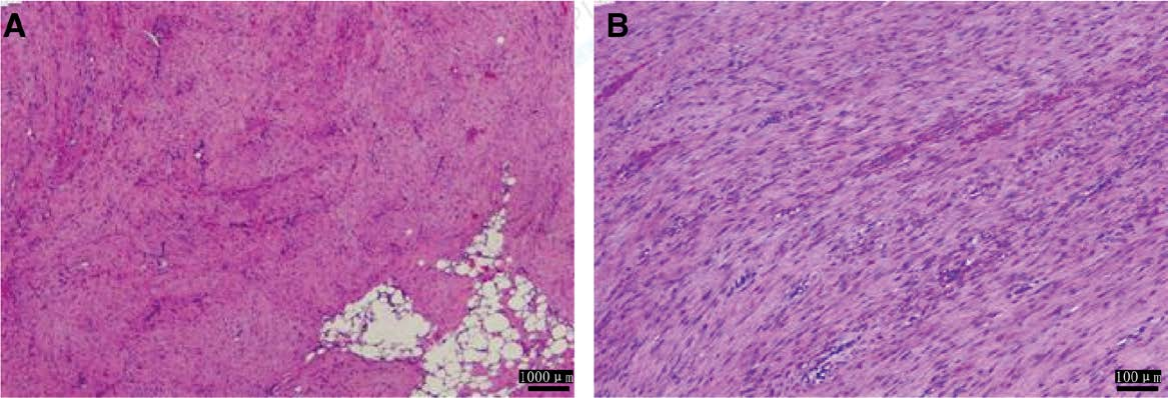
Histopathological and immunohistochemical analysis of the recurrent abdominal wall tumor in Case 2 (hematoxylin and eosin staining). (A) Low-power view (scale bar = 1000 μm) demonstrating the characteristic infiltrative growth pattern of desmoid-type fibromatosis. (B) High-power view (scale bar = 100 μm) reveals bland, spindle-shaped fibroblasts arranged in long, sweeping fascicles within a collagenous stroma. A rare mitotic figure is noted, consistent with the intermediate-grade nature of this lesion.These images were retrieved from the hospital’s electronic medical record system, and their resolution reflects the standard archival quality.

**Figure 9.**
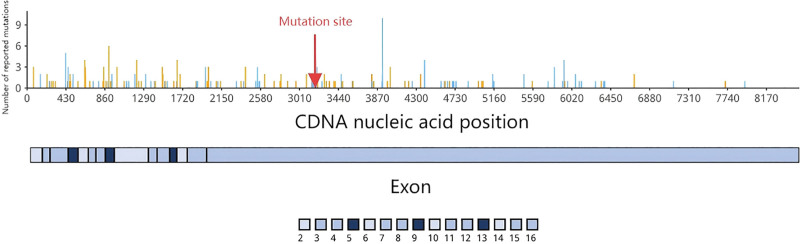
Sanger sequencing chromatogram of the APC gene in Case 2. The arrow indicates the heterozygous nonsense mutation c.3183_3187delACAAA (p.Gln1062Ter) in the tumor DNA. APC = adenomatous polyposis coli, DNA = deoxyribonucleic acid.

Contrast-enhanced CT at presentation identified a well-definedleft rectus abdominis mass (32 × 39 mm) and multiple mesenteric masses. These lesions demonstrated mild heterogeneous enhancement, less than that of the adjacent psoas muscle (Fig. 7). Management was tailored to each lesion. The symptomatic and progressive abdominal wall mass was completely resected with wide negative margins (R0 resection). In contrast, intraoperative exploration revealed that the multifocal mesenteric diseases were extensive, poorly demarcated, and involved critical mesenteric structures, making complete resection impossible without causing short-bowel syndrome. Therefore, a deliberate decision was made to refrain from resecting the mesenteric lesions to avoid high morbidity. This approach of selectively addressing symptomatic sites while preserving organ function is consistent with modern conservative management principles for DTs. Pathology confirmed intermediate-grade DT (Fig. 8). Immunochemistry: nuclear β-catenin (+), Caldesmon (+), SMA/DES (partial +), CD117/CD34/DOG1/S-100/SOX10/STAT6/ALK-1A4 (−), SDHB (+), Ki-67 ~ 15%. Genetic sequencing: no CTNNB1 mutation; heterozygous APC nonsense mutation c.3183_3187delACAAA (p.Gln1062Ter) (Fig. 9), causing frameshift and termination at codon 1062 via nonsense-mediated mRNA decay, confirming invasive fibromatosis.

#### 3.2.1. Follow-up

**Figure 10.**
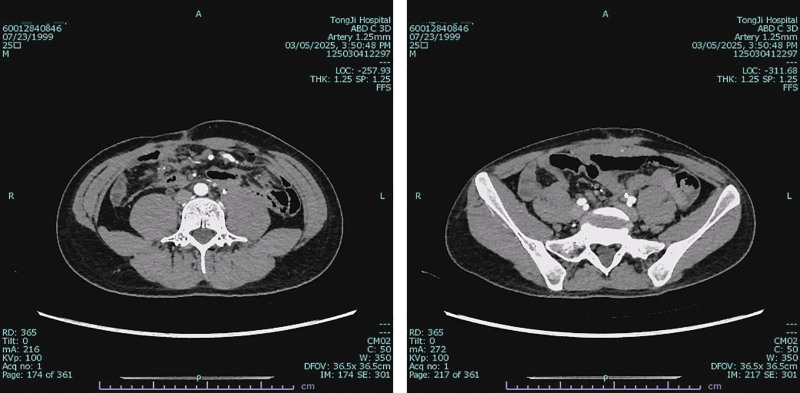
Case 2 patient’s follow-up CT image 3 years after surgery and initiation of systemic therapy. cDNA = complementary deoxyribonucleic acid, CT = computed tomography.

**Figure 11.**
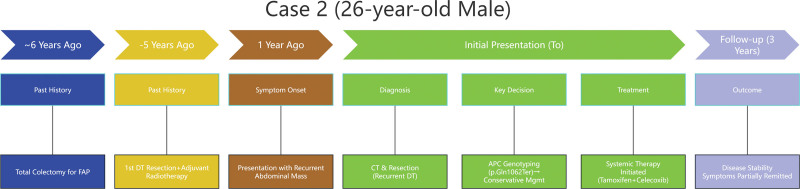
Timeline of the disease course in Case 2. CT = computed tomography, DT = desmoid tumor, FAP = familial adenomatous polyposis.

The identification of an intermediate-region APC mutation (codon 1062) supported a strategy of minimizing aggressive interventions. Following resection of the symptomatic abdominal wall lesion, the patient was started on low-intensity systemic therapy (tamoxifen and celecoxib) for the residual mesenteric disease by the multidisciplinary tumor board. The follow-up strategy is the same as Case 1, surveillance imaging at 6-month intervals: CT showed stable abdominal wall (16 × 8 mm) and right abdominal lesions (22 × 16 mm in 2023; 32 × 20 mm in 2025 [Fig. 10]). At a 3-year follow-up, disease stability confirmed, consistent with indolent APC codon 1062 mutation. Symptoms partially remitted. The timeline of the disease course has been listed (Fig. 11).

## 4. Discussion

Comprehensive APC genotyping establishes a risk-adapted stratification paradigm where intermediate-region mutations (codons 607/1062) mandate conservative management – prioritizing active surveillance for asymptomatic presentations and low-intensitysystemic therapy (e.g., nonsteroidal anti-inflammatorydrugs [NSAIDs]/antiestrogens) for symptomatic cases – thereby enabling therapeutic de-escalation through avoidance of unnecessary surgery or aggressive tyrosine kinase inhibitors (TKIs), while simultaneously providing enhanced prognostic precision that supersedes traditional clinicopathological factors in predicting spontaneous regression or disease stability.

### 4.1. Molecular pathogenesis

DT etiology remains incompletely defined, with risk factors including trauma, hormonal influences (e.g., pregnancy), and genetic predisposition.^[10]^ Pathogenesis is primarily driven by somatic APC or CTNNB1 mutations.

Sporadic DTs (80%–90%) typically harbor CTNNB1 mutations, predominantly at codons 41 (p.Thr41Ala) or 45 (p.Ser45Phe/Pro).^[11]^ FAP-associated DTs arise from APC mutations, with 3′-end mutations (e.g., codons 1286–1513) conferring higher incidence and aggressiveness.^[12,13]^

Nuclear β-catenin accumulation is a hallmark: mutant CTNNB1/APC proteins disrupt β-catenin degradation, enabling nuclear translocation. T cell factor/lymphoid enhancer factor family complex formation upregulates cyclinD1/c-Myc, driving proliferation. Notably, CTNNB1 and APC mutations are mutually exclusive in DTs, as established by international consensus.^[1]^ Consequently, CTNNB1 mutations are rarely identified in patients with FAP-associated DTs.

The profound impact of APC mutation location on DT behavior can be explained by the structure–function relationship of the APC protein. The APC gene encodes a large multidomain protein that plays a critical role in the β-catenin destruction complex. Truncating mutations occurring in different regions of the gene result in distinct functional consequences. Mutations in the 5′ end (e.g., before codon 158) often lead to very short, unstable protein products that may be rapidly degraded, resulting in a mild phenotype or even a reduced risk of DT development. In contrast, our cases highlight intermediate-region mutations (e.g., codons 607 and 1062). These truncations produce proteins that retain the N-terminal oligomerization domains but lack critical segments required for efficient β-catenin binding and degradation. This partial loss of function leads to moderate, dysregulated Wnt pathway activation, which clinically manifests as the indolent behavior observed in our patients – characterized by spontaneous regression or long-term stability. Conversely, 3′-end mutations (e.g., beyond codon 1400) produce longer, more stable truncated proteins that can act in a dominant-negative manner, more effectively sequestering components of the destruction complex and leading to robust β-catenin accumulation and a highly aggressive clinical course associated with persistent growth and higher mortality.^[13]^ This mechanistic understanding underscores why genotyping is not merely a prognostic tool but a fundamental guide to the biology of the disease, directly informing management intensity.

### 4.2. Diagnosis and assessment

DTs lack specific symptoms; ~50% present as painful abdominal masses because of obstruction, nerve compression, or hemorrhage. They exhibit slow growth, firm texture, and local invasiveness without metastasis.^[1]^

Diagnosis integrates clinical history, imaging, and histopathology. Imaging modality is location dependent: CT for intra-abdominal, magnetic resonance imaging for extra-abdominal,and ultrasound for superficial DTs.^[14]^

Genetic testing identifies CTNNB1 or APC mutations, confirming the diagnosis and enabling molecular subtyping of DTs. This molecular information is crucial for guiding treatment decisions and prognostic assessment.^[13,15]^ In our cases, APC genotyping (codons 607/1062) predicted indolent behavior, validated by spontaneous regression (Case 1) and disease stability (Case 2).

### 4.3. Treatment strategies and staging

To address the characteristics and clinical treatment challenges of DT, Church et al^[16]^ proposed a staging system incorporating clinical presentation, tumor size/location, and genetic/familial factors (Table 1), aiming to standardize treatment approaches and facilitate multicenter research. A correlation exists between APC mutation site and disease stage: mutations in the 5′ region are associated with stage I/II disease, typically exhibiting stable or regressing tumors. Intermediate-region mutations progress to stage III/IV in approximately 12% of cases. Mutations at the 3′ end correlate with stage III/IV disease in up to 44% of cases and a mortality rate of 17.4%.^[13]^ When integrated with the clinical staging system, mutation sites significantly influence both disease incidence and clinical aggressiveness, further underscoring the critical role of genetic sequencing in stratifying treatment strategies for DTs. In summary, treatment selection for DTs varies depending on the specific site of the APC gene mutation.

**Table 1:** Proposed treatment staging system for desmoid tumors (adapted from Church et al^[16]^).

Stage	Definition and criteria	Recommended treatment
I	Asymptomatic, stable tumor (usually small, incidentally discovered)	Observation or NSAIDs (e.g., sulindac)
II	Symptomatic and ≤10 cm, no rapid growth	Surgical resection (low-risk cases) or combined antihormonal therapy (tamoxifen, raloxifene + NSAIDs)
III	Symptomatic or 11–20 cm, or slow growth (<50% diameter increase/6 months)	Combination chemotherapy (vincristine/methotrexate) + antihormonal therapy; switch to tumor-specific chemotherapy if progression occurs
IV	Symptomatic and >20 cm, rapid growth, or life-threatening complications	Aggressive surgery (possible enzyme adjunct) or radiotherapy; doxorubicin/dacarbazine-based chemotherapy

NSAIDs = nonsteroidal anti-inflammatory drugs.

#### 4.3.1. Active surveillance

A significant proportion of DTs (up to 50%) follow an indolent course and may not require active intervention. Tumors demonstrating stability for over 1 year rarely necessitate treatment.^[12]^ Genetic sequencing at initial diagnosis, particularly in patients with FAP, is essential for identifying the mutation site. For patients harboring 3′-end mutations, which portend a more aggressive course, a conservative surgical approach is warranted, and prophylactic colorectal surgery may be delayed accordingly.^[13]^ Both the National Comprehensive Cancer Network guidelines and the Desmoid Tumor Working Group (DTWG) guidelines recommend active surveillance as the preferred initial strategy for patients without significant symptoms, irrespective of tumor size or location. The recommended surveillance protocol involves cross-sectional imaging (CT or magnetic resonance imaging) every 3 to 6 months following diagnosis to monitor tumor size. Progression is defined as a ≥20% increase in tumor diameter on 2 consecutive scans or significant worsening of symptoms.^[1,6]^

Furthermore, clinicians must be vigilant for serious complications such as NOMI, a recognized though rare complication in patients with mesenteric DTs exhibiting significant vascular involvement.^[17]^ NOMI can arise from direct mechanical compression or even a vascular “steal phenomenon” by the tumor. In our Case 1, the presence and subsequent resolution of NOMI-related radiological signs following tumor regression underscore the importance of close monitoring for such complications, even when pursuing a conservative strategy for indolent genotypes.

#### 4.3.2. Surgical intervention

Surgery is reserved for progressive tumors, severe symptoms, or high-risk morbidity (e.g., bowel obstruction), guided by location and morbidity. The National Comprehensive Cancer Network guidelines discourage first-line surgery due to comparable 5-year progression-free survival (PFS) (53% surgery vs 58% conservative). However, recurrence risk is substantial. Surgery requires multidisciplinary tumor board’s discussion.^[1,6]^ The DTWG guidelines limit priority to abdominal wall DTs with progression, severe symptoms, or functional impairment. Adjuvant radiotherapy shows no recurrence-free survival) benefit (relative risk: 0.69, 95% CI: 0.41–1.17) and may increase sarcoma risk.^[1]^

Recurrence rates remain high (23%–90%), especially in FAP-associated DTs. In patients with germline 3′ APC mutations, surgical trauma can trigger second-hit mutations, activating β-catenin and increasing aggressiveness.^[13]^ For 3′ APC mutations, delayed or minimally invasive surgery reduces recurrence risk.

#### 4.3.3. Systemic therapies

Systemic therapy is primary for DT patients ineligible for surgery/radiotherapy or with rapid recurrence, including cytotoxic chemotherapy, TKIs, hormonal agents, and NSAIDs.^[18]^ Cytotoxic chemotherapy (e.g., methotrexate/vinblastine and doxorubicin/dacarbazine) is preferred for severe symptoms requiring rapid shrinkage. For less urgent cases, consider TKIs or hormonal–NSAID combinations.^[19]^ International guidelines strongly recommend TKIs (e.g., sorafenib and pazopanib) as a cornerstone therapy for progressive DT.^[1,19]^ The DTWG guidelines prioritize sorafenib (objective response rate: 33%, improved PFS) and pazopanib (6-month PFS 82%) as preferred TKIs.^[1]^ TKIs are contraindicated with high gastrointestinal perforation risk or impaired drug absorption. Frequent estrogen receptor expression in FAP-associated DTs supports estrogen receptor-targeting agents (e.g., tamoxifen and raloxifene), often combined with NSAIDs.^[20]^ Testosterone or GnRH agonists (e.g., goserelin) are alternatives. Limited data suggest meloxicam may benefit CTNNB1 p.Ser45Phe mutation patients.^[19]^ The DTWG guidelines note limited high-quality evidence for hormonal therapies and NSAIDs. Thus, use requires individualized assessment and further clinical study.

#### 4.3.4. Emerging therapies

Given the limitations of surgery and ongoing debates surrounding systemic therapy selection, emerging local ablative techniques (e.g., cryoablation and radiofrequency ablation) are being explored. Current evidence remains limited to small case series, necessitating validation via larger long-term studies.^[21]^

### 4.4. Prognostic factors

Postoperative recurrence rates: 12%– to 23% overall, but 90% for FAP-associated mesenteric DTs.^[22]^ Established independent prognostic factors include tumor size, location, and patient age and gender. Younger age and female gender increase progression risk; older age predicts longer PFS.^[23]^ Regarding genetic predisposition, families carrying germline APC mutations in the 5′ region have a lower risk of developing DT.^[13]^

CTNNB1 p.Ser45Phe mutations predict rapid progression and high recurrence. Additional predictors: >20% nuclear β-catenin immunochemistry, Trisomy 8, and ADAM12/FAP-1α/WISP1 overexpression.^[11,12]^

APC mutation locus dictates prognosis:^[13]^

1. 3′ mutations (e.g., codon 1400): 21% persistent growth, 17.4% mortality;
2. 5′ mutations (e.g., codon 400): minimal symptoms, negligible mortality; and
3. intermediate mutations (e.g., codon 607/1062): 12% progression, 4.5% mortality.

Our cases validate this model: intermediate APC mutations (codons 607/1062) showed spontaneous regression (Case 1) and stability (Case 2), aligning with Church et al’s 12% progression risk. This confirms that APC site analysis enables conservative management.

### 4.5. Genotype-guided management advantages

These mesenteric DTs with intermediate-region APC mutations (codons 607/1062) demonstrated spontaneous regression or long-term stability, validating their low-progression-risk phenotype. Key implications include:

1. genotype–phenotype correlation: intermediate APC mutations predict indolent behavior, permitting active surveillance or low-intensity therapy (e.g., NSAIDs/antiestrogens);
2. therapeutic de-escalation: early mutation identification avoids unnecessary surgery/aggressive TKIs;
3. prognostic precision: mutational analysis surpasses traditional factors (e.g., size/location) in risk stratification.

### 4.6. Limitations of conservative management and contemporary alternatives

While our cases validate a genotype-guided conservative approach for indolent-phenotype DTs, it is imperative to recognize its boundaries and contextualize it within the broader therapeutic arsenal. Conservative management is not appropriate in scenarios of life-threatening or rapidly progressive disease. These include symptomatic bowel obstruction, ischemia (including the NOMI highlighted in our Case 1^[17]^), uncontrollable pain, or a documented rapid increase in tumor size (>50% diameter growth within 6–12 months).^[1,6]^ In such situations, more proactive interventions are warranted. Surgical resection, despite its historically high recurrence rates, remains a cornerstone for symptomatic, resectable lesions, particularly in the abdominal wall. Importantly, recent advances in minimally invasive (e.g., laparoscopic and robotic) techniques for peritoneal and mesenteric tumors may offer reduced morbidity compared to traditional laparotomy, as evidenced by emerging series,^[24]^ providing valuable alternatives when surgery is necessary. For inoperable, progressive disease not amenable to surgery, systemic therapies like TKIs (sorafenib and pazopanib) are strongly recommended by international guidelines due to their proven efficacy.^[1,18]^ Thus, the paradigm we advocate is not the replacement of these modalities, but rather the use of comprehensive genotyping at diagnosis to better stratify patients, ensuring that aggressive therapies are reserved for those with genuinely aggressive disease biology, while sparing others from unnecessary treatment-related morbidity.

### 4.7. Study limitations

This study has several limitations that should be considered when interpreting our findings. First, the conclusions are drawn from only 2 cases, which limits the generalizability of our results and the statistical power of the analysis. Although the genotype–phenotype correlation we observed is compelling and consistent with existing literature, it requires validation in larger, prospective cohorts. Second, as a retrospective case series from a single institution, the potential for selection bias cannot be excluded. The patients presented here were referred to a tertiary care center and had comprehensive genetic testing, which may not reflect the broader population of patients with DTs. Third, the assessment of tumor response was based on standard clinical and radiological criteria (RECIST), and we did not employ centralized imaging review or more advanced functional imaging techniques, which could provide additional insights. Furthermore, the resolution of some histopathological images is limited as they were retrieved from the hospital’s electronic medical record system, which is a common constraint in retrospective clinical studies. Nevertheless, all key diagnostic features remain discernible for expert review. Finally, while we demonstrate a strong clinical correlation between intermediate-regionAPC mutations and indolent behavior, our study does not provide direct experimental evidence to elucidate the underlying molecular mechanisms driving spontaneous regression. Future research integrating multi-omics approaches with clinical outcomes is needed to fully unravel these mechanisms.

## 5. Conclusion

APC mutation sites (e.g., intermediate codons 607/1062) predict DT behavior, evidenced by spontaneous regression without intervention in Case 1 and sustained stability under systemic therapy in Case 2. Comprehensive genetic sequencing is pivotal for individualized management, preventing overtreatment through genotype-driven stratification that prioritizes active surveillance as the initial strategy for most patients, reserves surgery for specific indications, and establishes TKIs as cornerstone pharmacologic intervention while evaluating hormonal agents/NSAIDs through individualized risk-benefit assessment.

Future large-scale studies must validate mutation-outcome correlations and elucidate molecular mechanisms governing spontaneous regression.

## Acknowledgments

We are also grateful to all the researchers, including the physicians, nurses, and technicians, who participated in this study.

## Author contributions

**Conceptualization:** Niu Huang, Xue-lai Luo.

**Data curation:** Guang Shi.

**Funding acquisition:** Xue-lai Luo.

**Investigation:** Guang Shi.

**Methodology:** Niu Huang.

**Project administration:** Niu Huang, Xue-lai Luo.

**Supervision:** Niu Huang, Xue-lai Luo.

**Validation:** Niu Huang.

**Writing – original draft:** Guang Shi.

**Writing – review & editing:** Niu Huang, Guang Shi, Xue-lai Luo.

## Correction

This article was originally published with an incorrect province in affiliation and thus the online version has now been updated from *“Department of Gastrointestinal Surgery, The People’s Hospital of Tongcheng, Tongcheng, Anhui Province, China”* to *“Department of Gastrointestinal Surgery, The People’s Hospital of Tongcheng, Tongcheng, Hubei Province, China.”*
